# P-721. Risk Factors Associated with COVID-19 Among Fully Vaccinated Healthcare Workers in Lima, Peru

**DOI:** 10.1093/ofid/ofae631.917

**Published:** 2025-01-29

**Authors:** Candice romero, Diana Ponce, Giselle Soto, Perrine Marcenac, Eva Leidman, Garrett W Mahon, Ashley Fowlkes, Joan M Nayra

**Affiliations:** Naval Medical Research Unit SOUTH, Lima, Lima, Peru; Naval Medical Research Unit SOUTH, Lima, Lima, Peru; Naval Medical Research Unit SOUTH, Lima, Lima, Peru; US CDC, Lima, Lima, Peru; US CDC, Lima, Lima, Peru; Centers for Disease Control and Prevention, Atlanta, Georgia; Centers for Disease Control and Prevention, Atlanta, Georgia; Naval Medical Research Unit SOUTH, Lima, Lima, Peru

## Abstract

**Background:**

Healthcare workers (HCWs) are an important target group for COVID-19 vaccination. In February 2021, Peru launched a COVID-19 vaccination campaign among HCWs using a BBIBP-CorV inactivated whole-virus vaccine (Sinopharm). We aim to understand risk factors for SARS-CoV-2 infection after vaccination using a cohort of HCWs in two hospitals in Lima, Peru.

**Methods:**

Between August 2021-June 2022, HCW who received at least two COVID-19 Sinopharm vaccine doses were enrolled in the cohort and followed for 6 months. Participants provided a self-collected nasal swab weekly for SARS-CoV-2 by reverse transcriptase polymerase chain reaction testing. SARS-CoV-2 infection was confirmed by a positive PCR test result at least 14 days after receiving the second vaccine dose. Adjusted hazard ratios (aHR) were used to identify associations with infection accounting for potential confounding. Person-time was calculated based on days between negative PCR results, capped at 10 days; in cases of infection, the time between positive and negative tests was halved, with half the days considered at risk.

**Results:**

Of 670 vaccinated HCWs, 111 (16.6%) tested positive for SARS-CoV-2 during the follow-up period. In adjusted models, prior report of COVID-19 illness in the previous 12-months was associated with reduced risk of infection (aHR: 0.98 [95% CI:0.97‒0.99], p=0.026). Being beyond 6 months since the last vaccination was significantly associated with an increased risk of infection (aHR: 3.22 [95% CI:1.66‒6.23], p=0.0001) as well as working in an outpatient setting (aHR: 1.94 [95% CI:1.06‒3.53], p=0.031).

**Conclusion:**

In this cohort of healthcare workers undergoing regular asymptomatic COVID testing, our findings suggest evidence of waning protection at 6 months post-vaccination BBIBP- CorV. Additionally, preliminary indications suggest that healthcare worker behaviors or specific occupational exposures may contribute to increased infection risk even following vaccination. In addition, these results support the inclusion of HCWs as a priority group for additional booster doses of COVID-19 vaccine at 6 months after the most recent dose.

**Disclosures:**

**All Authors**: No reported disclosures

